# Current Advances on the Development and Application of Probiotic-Loaded Edible Films and Coatings for the Bioprotection of Fresh and Minimally Processed Fruit and Vegetables

**DOI:** 10.3390/foods10092207

**Published:** 2021-09-17

**Authors:** Kataryne Árabe Rimá de Oliveira, Karina Felix Dias Fernandes, Evandro Leite de Souza

**Affiliations:** Laboratory of Food Microbiology, Health Sciences Center, Department of Nutrition, Federal University of Paraíba, João Pessoa 58051-900, Paraíba, Brazil; katarynearabe_@hotmail.com (K.Á.R.d.O.); kfdfernandes@gmail.com (K.F.D.F.)

**Keywords:** probiotic, biopolymers, biopreservation, antimicrobials, quality parameters, fruit, vegetables

## Abstract

The application of probiotics has emerged as an innovative bioprotection technology to preserve fresh and minimally processed fruit and vegetables. This review discusses the most recent advances on the development and application of probiotic-loaded edible films/coatings as a strategy to preserve fresh or minimally processed fruit and vegetables. Available studies have shown a variety of materials, including hydrocolloids (polysaccharides and proteins) and lipids, used alone or in combination to formulate edible films/coatings loaded with probiotics. Plasticizers and surfactants are usually required to formulate these edible films/coatings. The reported antimicrobial effects of probiotic-loaded edible films/coating and quality parameters of coated fruit and vegetables could vary according to the characteristics of the materials used in their formulation, loaded probiotic strain and its dose. The antimicrobial effects of these films/coatings could be linked to the action of various metabolites produced by embedded probiotic cells with inhibitory effects on microorganisms contaminating fruit and vegetable surfaces. The implication of the use of probiotic-loaded edible films/coatings should be their antimicrobial effects against pathogenic and spoilage microorganisms and efficacy to control the ripening of fruit and vegetables, helping the coated products to maintain their safety, quality, nutritional and functional characteristics for a more prolonged storage period.

## 1. Introduction

The demand of consumers for non-chemically preserved, high-quality and healthy foods has been continuously increasing, where fresh or minimally processed fruit and vegetables have gained preference due to their high nutritional value, overall good acceptance and association of their consumption with different health benefits [1]. However, the quality and safety of fruit and vegetables typically decrease during the postharvest processing due to their high moisture content, ripening, senescence, microbial growth and environmental factors. The minimal processing may also cause physical damage and enhance the ethylene production in fruit and vegetables, accelerating their ripening and senescence [2]. These factors increase the postharvest losses in fruit and vegetables, which can reach up to 25–40% before the final consumption [3,4].

Most of the traditional preservation methods applied to fruit and vegetables are based on the control of transpiration and respiration rate and microbial spoilage. However, these preservation methods have some practical limitations, to cite: (i) the extensive pre- and postharvest use of agrochemicals has been linked to the development of resistant fungal strains, besides increasing the amounts of toxic residues in the final products [5]; (ii) the prolonged storage period under low temperatures enhances the cold damage and induces undesirable physiological alterations in fruit and vegetables [6]; and (iii) the thermal processing can lead to loss of sensory and nutritional characteristics in fruit and vegetables [3]. The development of alternative methods to guarantee the microbial safety and improve the postharvest quality characteristics of fruit and vegetables has been a research focus, but some of the new developed technologies (e.g., microwave, electric pulsed field, ultraviolet radiation and high pressure) have been also linked to the rising of undesirable effects on the final product quality [3]. Considering these aspects, the application of probiotics, which are found naturally in various foods, has emerged as an innovative technology to postharvest bioprotection of fresh and minimally processed fruit and vegetables [7,8].

Bioprotection with the use of beneficial microorganisms could be effective to control the presence of pathogenic and spoilage microorganisms through the production of specific metabolites, which can modify the pH, moisture and nutrient dynamics in a fruit or vegetable of interest [1]. The application of probiotics could prolong the storability of fruit and vegetables without the use of chemical antimicrobial treatments. However, the direct application of probiotics to the surface of fruit and vegetables may adversely affect some of the physical or sensory properties of these products, negatively influencing their acceptance by consumers and even decreasing the survival of added probiotic during storage [9].

The use of edible films and coatings has been considered a possible solution to overcome the limitations related to the compromised action of probiotics in fresh and minimally processed fruit and vegetables [10]. Edible films/coatings are bidirectional strategies to preserve fruit and vegetables causing improvements in safety and protecting the sensory and nutritional qualities of coated products, besides acting as carriers for probiotic delivery [11,12]. Suitable edible matrices to carry probiotics must enable higher probiotic survival rates during storage and consumption, better control of probiotic loaded doses and easier and affordable possibilities for application of these microorganisms [10].

However, the incorporation of probiotics into edible films/coatings also brings some challenges since the probiotic loading should not negatively affect the mechanical and barrier properties of the formulated films/coatings and probiotic cells should remain adequately distributed in the formulated films/coatings, as well as exert the expected preservation effects when applied to the selected foods [10]. Additionally, these probiotic-loaded films/coatings should neutralize the reported disadvantages related to the direct application of probiotics and help the carried probiotics to reach the human gut in sufficient doses to exert a claimed health effect on the host, adding value to the coated fruit or vegetable [13,14].

Due to the fact of being consumed together with the food, probiotic-loaded edible films/coatings must meet some regulatory specifications, which are individually evaluated based on the formulations and selected applications [10]. These films/coatings must meet the legislation requirements of the country where they are produced, featuring the eating quality, safe materials and production following good manufacturing practices [10,15]. In general, all ingredients added to foods for human consumption must be harmonized with generally recognized as safe (GRAS) regulations of the Food and Drug Administration (FDA) or with presumption of safety status (QPS) of the European Food Safety Authority (EFSA) [16].

The first study investigating the incorporation of probiotics into edible coatings for application on fruit was performed by Tapia et al. [17], evaluating the use of alginate and gellan composite material loaded with *Bifidobacterium lactis* subsp. *lactics* Bb-12 to coat apple and papaya chips. Since then, other investigations have been carried out to evaluate the efficacy of loading different probiotic strains into edible films/coatings to confer functional or antimicrobial properties when applied to fresh or minimally processed fruit and vegetables. In general, the number of studies approaching the application of probiotic-loaded edible films/coatings in fruit and vegetables has been much lower than that of studies including only experiments with laboratory media.

This review aimed to summarize and discuss the most recent advances on the development and application of probiotic-loaded edible films/coatings to preserve fresh and minimally processed fruit and vegetables, with focus on the main materials used for the formulations of these composite materials, their effects on probiotic survival and impacts of their application on different parameters related to the quality, safety and storability of the coated fruit and vegetables.

## 2. Materials Used to Formulate Probiotic-Loaded Edible Films/Coatings for Application to Fresh and Minimally Processed Fruit and Vegetables

Edible films/coatings can improve the food quality and safety, strengthening the food’s natural layers and creating a semipermeable barrier to water vapor, gases and transfer of soluble material from coated food [9]. Edible films/coatings could also meet environmental concerns since they are formulated typically with biodegradable, biocompatible, low-toxicity and GRAS/QPS materials. Additionally, formulated edible films/coatings should have good barrier and mechanical properties and physicochemical and microbiological stability, in addition to being of low cost and sensorially and functionally compatible with the food to be coated [10].

Although the terms edible films and coatings have been sometimes used as synonyms, each of them represents a different concept. An edible coating is characterized as a thin layer of material suitable for consumption and formed by suspensions of hydrocolloids or food-grade lipids applied to the food surface by spraying, smearing or dipping, and after drying it forms a thin layer on coated food. An edible film is characterized as a thin layer of edible material obtained from food-grade filmogenic suspensions and formed on the food surface as a coating or placed preformed on food surfaces [3]. These coating technologies decrease fruit and vegetable respiration due to the formation of a barrier to water vapor, oxygen and carbon dioxide, providing a high-humidity environment [3,18].

The entrapment of probiotics into edible films/coatings has been typically done with a direct method where the probiotic cells (cell-free supernatants or purified metabolites) are incorporated into a film/coating-forming solution just after its production [19]. Edible films/coatings are mainly formed through wet and dry processing. In the wet processing (also referred as solvent casting), the biopolymers and other additives are dissolved in solvents (usually water and ethanol) and the film/coating-forming solution is provided after homogenization and evaporation of the solvent. This method is ideal for coating applications in fluid mode, brushing, dipping or spraying completely onto the food. The dry processing is based on the thermoplastic behavior of some proteins and polysaccharides at low moisture levels in pressing molding and extrusion [10,11,19]. Despite the application method, it is important to produce a film/coating with good water vapor barrier properties to retard fruit and vegetable surface dehydration, especially in fresh products, as well as with good gas control properties, especially for oxygen and carbon dioxide, to retard respiration and oxidation processes [10].

Edible films/coatings can be formulated using materials with film-forming properties classified into three categories: hydrocolloids, lipids and composites [19]. Hydrocolloids (polysaccharides and proteins) and lipids have been the materials most used to formulate edible films/coatings, with polysaccharides being the easiest to purchase and most suitable to this end [11]. These macromolecules represent the basis of films/coatings, being dissolved in a solvent to form a cohesive set. The cohesion properties have been linked to the polymer characteristics, such as the molar mass, polarity and chain structure [10]. Other materials, such as plasticizers, surfactants, crosslinkers and emulsifiers, have also been used to prepare film/coating solutions to enhance their stability and/or mechanical properties (e.g., flexibility, barrier and optical characteristics) [15]. The materials used to formulate probiotic-loaded edible films/coatings applied to fresh or minimally processed fruit and vegetables in different studies are shown in Table 1.

Polysaccharide-based probiotic-loaded edible films/coatings have caused minor odor impacts on coated products, besides having good mechanical properties and oxygen and oil barrier effects. However, the higher moisture permeability due to their hydrophilic properties has been an important disadvantage to these composite materials [19]. Cellulose derivatives, inulin, alginate, starch derivatives, pectin derivatives, seaweed extracts and chitosan have been the polysaccharides most used to formulate probiotic-loaded edible films/coatings [18,25,28,29].

Some studies have investigated the use of alginate as a material to carry probiotics, being cited as a strategy to improve the quality, functionality and storage stability of fresh and minimally processed fruit and vegetables [21,22,29]. The immersion of fruit and vegetables in a calcium chloride solution after the alginate dispersion application has been necessary to induce a gelling mechanism and crosslinking reactions to form the coating [30]. Additionally, alginate coatings have shown better performances than chitosan coatings in fruit, not affecting the survival of probiotics during storage [18]. Carboxymethylcellulose coatings have been shown to be effective to carry and deliver adequate doses of probiotics on fresh strawberries, improving the physicochemical and microbiological characteristics during refrigeration storage [25].

Protein-based probiotic edible films/coatings are generally formed from protein solutions/dispersions as the solvent (ethanol, water or their mixture) evaporates. Protein-based films/coatings typically have poor water resistance, but they possess better mechanical and barrier properties, allowing the formation of less porous and more compact structures [31]. Gelatin, collagen, casein and whey protein have been the proteins most used for probiotic-loaded edible film/coating formulation. The use of proteins has commonly increased the probiotic survival in edible films/coatings via scavenging free radicals and conveying micronutrients, such as peptides and amino acids, helping the probiotic cells to resist the film/coating processing and storage [19,25].

Gelatin-based films have shown a superior protective effect on the survival of different probiotics during the film drying and storage when compared to low-methoxylpectin films [25]. Sodium caseinate-based coatings were reported as adequate sources of nutrients to keep high viable counts of probiotic *Lactiplantibacillus plantarum* [26]. Probiotic *Lacticaseibacillus casei* kept recommended viable counts (≥6 log CFU/g) in whey protein isolate films during 14 days of refrigeration storage [23].

Lipid-based edible films/coatings are good barriers to moisture transfer due to their low polarity, but these composite materials have typically weaker gas permeability and mechanical properties compared to polysaccharide and protein-based edible films/coatings [10,11]. Lipids are difficult to apply on the surface of some foods due to their poor adhesion to hydrophilic surfaces [32], which makes necessary their combination with other materials (e.g., proteins and polysaccharides) to improve specific characteristics of formulated films/coatings [15]. Vegetable oils, natural waxes, acetoglycerides, resins and fatty acids have also been used to formulate edible films/coatings. The use of sunflower oil to formulate probiotic-loaded films/coatings has resulted in enhanced water barrier characteristics in these materials [21,27].

The combination of two or three different substances could be used to formulate edible films/coatings in order to improve the physicochemical and mechanical properties of a specific structural component with benefits from a synergistic action of the combined heterogeneous materials. This combination could allow the development of films/coatings with superior efficacy and increased application possibilities since each substance has unique and limited functions [15,21]. The successful development of multi-component films/coatings depends on the compatibility of the substances used for their formulation [33]. For example, the interaction of anionic compounds with cationic protein-based polymers could form insoluble complexes, while neutral protein aggregates could form soluble complexes [33].

Microbial biopolymers, especially exopolysaccharides, have been used to formulate probiotic-loaded edible films/coatings. These biopolymers are produced primarily by lactic acid bacteria (LAB) and used to enhance the texture of foods and their health benefits, as well as to inhibit the growth of pathogenic and spoilage microorganisms [33,34]. Gum-like exopolysaccharides can protect LAB and act as a carbon supply for these microorganisms. Furthermore, exopolysaccharides from LAB usually have good film-forming properties, enabling their use to form the film/coating structure and exploitation as additives in probiotic-loaded films/coatings [33].

Plasticizers are usually required to formulate edible films/coatings, particularly in combination with polysaccharides or proteins. Plasticizers are low molecular weight and usually hydrophilic compounds able to decrease the glass transition temperature and increase the toughness, flexibility and tear resistance of edible films/coatings [17,20]. The selection of a plasticizer for edible film/coating formulation should consider the adaptability and persistence of the plasticizer, as well as the required physical characteristics for the developed film/coating. Glycerol has been the plasticizer most frequently used to formulate films/coatings to be applied on fruit and vegetables [2,20,23,28], providing increased moisture content and gas permeability to these structures when compared to other plasticizers (e.g., sorbitol) [35].

Surfactants, such as oleic acid and polysorbate 80, have been incorporated into probiotic-loaded biopolymer-based edible films/coatings to enhance their adherence to fruit and vegetable surface. Addition of oleic acid had no clear effect on the probiotic survival in protein-based films/coatings, although it enhanced the probiotic viable counts on grapes treated with a probiotic-loaded starch coating during 7 days of room temperature storage [26].

Despite the reported benefits on coated fruit and vegetables, some physicochemical (e.g., permeability, thickness and opacity/transparency) and mechanical properties (e.g., flexibility, tensile strength, toughness and elongation at break, elasticity) of edible films/coatings can be modified with probiotic loading. These properties should be analyzed simultaneously for the design of appropriate edible films/coatings, providing an environment that guarantees the viability of the probiotics and efficacy of these composite materials to preserve fresh and minimally fruit and vegetables [11,19]. The incorporation of *L. plantarum, Pediococcus pentosaceus*, carboxymethylcellulose and glycerol resulted in cassava starch films with good appearance, degradability and lower permeability to water vapor, as well as with a compact and homogeneous structure, indicating uniform distribution of LAB cells [28]. The incorporation of *L. casei* resulted in whey protein isolate-based films with a yellowish hue, thicker structure, higher water solubility and resistance and lower flexibility. However, the *L. casei* loading did not affect the density and water vapor permeation of protein isolate films [23].

The optimization of the film/coating composition and processing is indeed very important for a successful application of the developed composite material, which must be tailored considering the practical application purpose and properties of the fruit/vegetable to be coated [10].

## 3. Probiotics Loaded into Edible Films/Coatings for Application to Fresh and Minimally Processed Fruit and Vegetables

The advances in technologies to develop edible films/coatings have allowed the enhancement of the functionality of coated foods with addition of nutrients, antioxidants, vitamins, minerals, prebiotics, probiotics and/or antimicrobial agents as adjuvants in the formulation of these composite materials [10,12].In particular, the demand for the consumption of foods containing probiotics has progressively increased in recent years due to the consumer concerns related to the adoption of healthy diets. Probiotics are defined as “live microorganisms that, when administered in adequate amounts, confer health benefit to the host” [36].

The main properties of probiotic strains are the resistance to gastrointestinal conditions and ability to adhere to human epithelial cells or mucus, improvement of the intestinal barrier integrity, enhancement of the immunological responses and reduction of the blood cholesterol levels in the host, besides exerting antimicrobial effects against pathogenic microorganisms through the production of antimicrobial substances and/or competition for growth factors, nutrients and/or binding sites [11,16,36]. Probiotics have also reduced the severity of diarrhea episodes, prevented intestinal inflammation and allergies and controlled genital urinary tract infections [9]. The effects of probiotics are strain-specific, and it is necessary to safely specify the genus, species and strain of the probiotic added or applied to a food [10].

Fermented dairy products have been the most traditional carriers for probiotics. However, other food matrices, such as fruit and vegetables, have been considered innovative and emerging matrices to deliver probiotics, mainly due to the increased interest of lactose-intolerant consumers or vegetarians in consuming these microorganisms [15]. As an alternative, probiotics could be incorporated into edible films/coatings to be applied to fresh or minimally processed fruit and vegetables, where these microorganisms exert antimicrobial effects on pathogenic and spoilage microorganisms, helping to improve the safety and prolong the storability of these foods [12].

LAB represent the major group of probiotic bacteria used by the food industry [28]. LAB are characterized as Gram-positive, rod-shaped, non-spore-forming, catalase-negative, acid-tolerant, aero-tolerant and strictly fermentative microorganisms, being GRAS and receiving the QPS [37]. Bifidobacteria have also commonly been used in probiotic food formulations, being characterized as Gram-positive, rod-shaped and strictly anaerobic microorganisms [38].

A variety of LAB have been used to formulate strategies to preserve fruit and vegetables through the addition of bioprotective cells, cell-free supernatants or purified metabolites incorporated into edible films/coatings. *Lacticaseibacillus rhamnosus*, *L. plantarum*, *Lacticaseibacillus casei*, *Lactobacillus acidophilus* and *Bifidobacterium animalis* subsp. *lactis* have been the LAB members most commonly studied for incorporation of probiotics into edible films/coatings to be applied on fresh/fresh-cut fruit and vegetables (Table 1).

The most important considerations for the successful use of probiotics for health promotion should be the ability of these microorganisms to keep their viability and metabolic activities. Although the maintenance of these characteristics is of importance during the passage throughout the gastrointestinal tract to set probiotics in the colon, the same importance is given to the extended survival and distribution of probiotics as bioprotective cultures in edible films/coatings for the postharvest preservation of fruit and vegetables [3,39,40].

### Survival of Probiotics in Edible Films/Coatings

Despite the potential benefits of the incorporation of probiotics into edible films/coatings to preserve fruit and vegetables, there are still some limitations for the efficacy of their use related to the survival of probiotics entrapped in these composite materials. The concentration of probiotic cells entrapped in edible films/coatings may suffer large variations during the film/coating preparation, storage condition and gastrointestinal digestion, which could negatively affect their food preservative and health-related biological properties [2,10,11].

The viable counts (survival) of probiotics loaded into edible films/coatings applied to fruit and vegetables found in different studies are shown in Table 2. Different intrinsic and extrinsic factors could affect the behavior and survival of probiotics within different food environments. Survival of probiotics depends on the type of the selected culture/strain, physiological state of probiotic cells, food matrix characteristics (e.g., pH and water activity), storage conditions, presence of protective carriers, oxygen and processing technologies [15,25]. According to the Food and Drug Administration and European Food Safety Agency, the minimum probiotic concentration required at the moment of consumption to obtain health benefits must be of ≥6 log CFU/g or mL of product [10,29].

A gelatin coating did not cause negative impacts on the viable counts of probiotic B. lactis in apple pieces after 10 days of refrigeration storage, while an alginate coating caused a decrease in viable counts during refrigeration storage [13]. The refrigeration storage has been the most used condition because of its lower impacts on the survival of probiotics in edible films/coatings. *L. acidophilus* La-14 kept viable counts of >7 log CFU/g into an alginate-based coating applied to carrot slices during 19 days of refrigeration storage [21].

**Figure 1:**
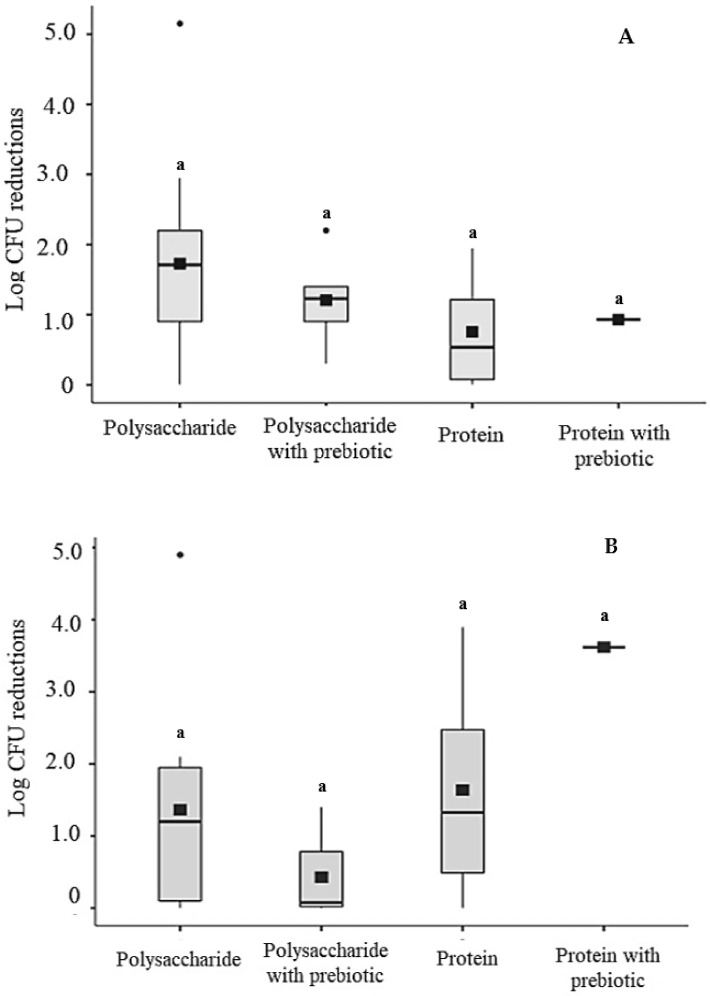
Assumed average variation (± standard deviation) of the reduction in initial viable counts of probiotics loaded into edible films/coatings. Columns with the same letters did not differ significantly (*p* > 0.05) based on Kruskal–Wallis test. Relative log CFU reduction is the difference between the population of probiotics loaded into films/coatings before and after (day zero or 1) application to fruit and vegetables (**A**) and at the end (last day) of the measured storage period (**B**).

Based on the quantitative analysis of different studies compiled in this review, which are partially presented in Table 2, it was possible to find that the viable cell counts of probiotics loaded into edible films/coatings (initial bacterial inoculum) typically differ from those found over the period that these composite materials are in contact with fruit and vegetables, i.e., at the beginning and end of the measured storage period (Figure 1). As shown in Figure 1A, reductions in viable counts (log CFU) have been found right after the application of the edible films/coatings on fruit and vegetables in a manner depending on the material used to formulate these structures. Lower average reductions in probiotic viable counts have been found in protein-based edible films/coatings, while higher reductions have been found in polysaccharide-based edible films/coatings with or without prebiotics (Figure 1A). Differences between the average log reductions of probiotic viable counts during storage of coated fruit and vegetables have ranged from zero to 4.9 log CFU (Figure 1B), where the average reductions also varied with the type of matrix used to prepare the edible films/coatings. The average reductions of probiotic viable counts during storage have been lower in polysaccharide-based edible films/coatings with and without prebiotics (0.43 ± 0.61 and 1.36 ± 1.45 log CFU, respectively). The variation in viable count reductions of probiotics loaded into edible films/coatings could indicate the necessity to explore more refined analyses to evaluate different influential factors (e.g., method of film/coating application and storage conditions) on the survival of probiotics entrapped in edible films/coatings applied to fruit and vegetables.

In addition to the survival capacity of probiotics in a food matrix during the processing and storage, the capability of these microorganisms of overcoming the stressful conditions found during the gastrointestinal passage should also be considered to ensure the desired physiological functionalities [20]. Probiotic *L. rhamnosus* and *B. lactis* loaded into alginate coatings applied to ready-to-eat apple were capable of resisting the exposure to simulated gastrointestinal conditions, keeping high viable counts (7.8 and 8.0 log CFU/g, respectively) [29]. *L. casei* LC-01 loaded into alginate coating applied to fresh-cut yacon kept high survival rates during exposure to simulated gastrointestinal conditions, with viable count reductions of approximately 2.9 log CFU/g [20].

Prebiotics have also been used to increase the survival of probiotics during the processing of edible films/coatings [11]. The incorporation of prebiotics into probiotic-loaded edible films/coatings has caused positive effects on the microstructure and stability of immobilized probiotic cells [41]. However, differences in coated fruit/vegetable composition, processing operations, storage conditions and probiotic inoculum level could influence the effects of the prebiotic addition on the survival of probiotics in formulated films/coatings [29].

Food ingredients must have some characteristics to be considered as prebiotics targeting the intestinal microbiota and related health benefits, to cite: resistance to gastric acidity, hydrolysis by mammalian enzymes and absorption in the upper gastrointestinal tract; fermentation by intestinal microflora; and selective stimulation of the growth and/or activity of intestinal bacteria associated with health benefits to the host [38]. However, the use of prebiotics in combination with probiotics could lead to changes in physical, chemical and biological properties of the formulated edible films/coatings, which deserve a careful evaluation during the development and application of these composite materials. Inulin, oligofructose and fructooligosaccharides (FOS) are the prebiotics most studied to formulate probiotic-loaded edible films/coatings to be applied to fruit and vegetables [12,20,22,29].

The incorporation of FOS into probiotic-loaded edible films/coatings can exert a plasticizer effect and contribute to protect probiotic cells during dehydration and storage [19]. The presence of inulin was shown to increase the survival of probiotic *L. rhamnosus* into gelatin-coated strawberries during 15 days of refrigeration storage [12]. The addition of inulin and oligofructose in an alginate-coating helped to keep the viable counts of probiotic *L. rhamnosus* above 6 log CFU/g on fresh blueberries during 21 days of refrigeration storage, while the viable counts in fresh blueberries treated with alginate coating without the prebiotics remained above 6 log CFU/g for only up to 7 days of refrigeration storage [23].

Other methodological variations can cause protective effects on probiotics in edible films/coatings. Sorbitol or similar compounds act as protective agents for microbial cells during drying or storage under low-moisture conditions [19]. The addition of some nutrients has also improved the survival of probiotics in edible films/coatings. The incorporation of coconut water into an alginate edible coating increased the survival of probiotic *L. acidophilus* during 7 days of refrigeration storage, with 3-log higher viable counts when compared to alginate coating without coconut water. The viable counts of *L.* *acidophilus* were above 6 log CFU/g in alginate + coconut water coating applied to carrots after 21 days of refrigeration storage [27].

The encapsulation of probiotics has been considered a strategy to increase the probiotic survival in edible films/coatings. *L. rhamnosus* B-445 microencapsulated by spray drying and loaded into an alginate + prebiotic inulin coating kept viable counts above 7 log CFU/g when applied on minimally processed apples during 13 days of refrigeration storage, while non-encapsulated *L. rhamnosus* B-445 had 2-log lower viable counts in the coating during storage [24]. Microencapsulation in a suitable and biodegradable matrix could protect probiotic cells against unfavorable environmental conditions, resulting in higher survival rates for a more prolonged period and improved preservation and functionality of coated fruit and vegetables [3].

## 4. Antimicrobial Effects of Probiotic-Loaded Edible Films/Coatings Applied to Fresh and Minimally Processed Fruit and Vegetables

Considering the ability to inhibit pathogenic microorganisms as one of the most important functional properties of probiotics [9,37], the loading of probiotics into edible films/coatings could be exploited as a strategy to increase the safety and stability of fruit and vegetables, especially to control postharvest diseases [10,11]. Probiotic bacteria typically produce bacteriocins, peptides, organic acids (acetic and lactic acid), hydrogen peroxide and diacetyl (protein-derived compound), which could help to control pathogenic and spoilage microorganisms, as well as the rot development in fruit and vegetables. Probiotic could also cause competition for resource consumption (e.g., vitamins, minerals, trace elements and peptides) with pathogenic and spoilage microorganisms co-existing in the same environment [9,12].

Therefore, the achievement of the antimicrobial efficacy involves a complex synergistic interaction between the direct action of probiotics on target microorganisms and the production of probiotic-derived metabolites under controlled conditions [3]. Still, the type and population of target microorganisms in coated fruit and vegetables also affect the achievement of the desired probiotic-mediated antimicrobial effects. An inverse correlation between the counts of probiotic *L. plantarum* with the counts of yeasts and molds was found on strawberries treated with probiotic-loaded carboxymethylcellulose coatings during refrigeration storage [25].

The antimicrobial effects of probiotics linked to bacteriocin production could be affected by the nature of the edible films/coatings [19], as well as by the load of the target microorganisms and their sensitivity to the produced bacteriocin [10]. Earlier studies reported greater production of bacteriocins by some LAB in polysaccharide-based media rather than in protein-based media, as well as a correlation between bacteriocin production rate and measured antimicrobial effects [42,43]. Bacteriocins have shown stronger inhibitory effects against Gram-positive bacteria, whereas organic acids act against Gram-negative bacteria [22]. High antimicrobial effects have been also attributed to LAB-derived peptides, which could act on target microorganisms through interactions with cell membrane components or internal targets related to DNA, RNA or proteins synthesis [3].

The antimicrobial effects of probiotic-loaded edible films/coatings when applied to fruit and vegetables in different studies are shown in Table 3. In addition to evaluating the effects of probiotic-loaded films/coatings on the autochthonous microbiota of fruit and vegetables [2,6,18], some studies have also used artificial contamination with pathogenic (e.g., *Listeria innocua* and *Escherichia coli* O157:H7) [22,29] or spoilage microorganisms (e.g., *Botrytis cinerea*) [26].

Freshly processed kiwi fruit coated with konjac gum and *L. plantarum* strains had reduced counts of molds and yeasts than uncoated fruit after 5 days of refrigeration storage [2]. Gelatin-based coating with *L. rhamnosus* and inulin reduced the counts of molds and yeasts and mesophilic bacteria in strawberries during 16 days of refrigeration storage [12]. An alginate coating loaded with *L. rhamnosus* and *B. lactis* had bactericidal effects against *L. innocua* and *E. coli* when applied to apple cubes, where the presence of *E. coli* and *L. innocua* did not affect the survival of *L. rhamnosus* and *B. lactis* in alginate coatings [29]. A sodium alginate coating loaded with *L. acidophilus* La-14 decreased the counts of molds and yeasts in minimally processed carrots during 19 days of refrigeration storage [21]. The application of coatings formulated with pregelatinized potato starch or sodium caseinate loaded with *L. plantarum* decreased the severity of rot caused by *B. cinerea* in grapes during cold storage, which was associated with the inhibitory effects of organic acids on target fungi [26].

Possible variations in antimicrobial efficacy of probiotic-loaded edible films/coatings could be related to the material used for probiotic entrapment, which could affect the permeability of edible films/coatings to the antimicrobial metabolites produced by probiotics, as well as the ability of the composite structures to protect and maintain probiotic active cells [10]. The temperature, processing conditions, pH alterations, enzymatic degradation and interactions with food ingredients could also affect the antimicrobial efficacy of probiotic-loaded edible films/coatings during storage [10]. A corn starch + gelatin coating containing irradiated (3.5 KGy) *L. paracasei* supernatant was more effective to reduce the percentage of rot and bacterial load in tomato during 14 days of refrigeration storage when compared to coating with non-irradiated supernatant. This result was attributed to a possible effect of gamma irradiation causing increases in antimicrobial activity of the supernatant since it doubled the bacterial count reductions when compared to non-irradiated supernatant [6].

## 5. Effects of Probiotic-Loaded Edible Films/Coatings on Quality Parameters of Fresh and Minimally Processed Fruit and Vegetables

The effects of probiotic-loaded edible films/coatings on the overall quality and sensory characteristics of fruit and vegetables are important aspects to be considered in a practical point of view [22]. Specifically, metabolically active probiotic cells entrapped in edible films/coatings commonly continue to produce organic acids or other metabolites over time, which could affect the sensory characteristics of coated fruit and vegetables [14,44].

Negative effects of the application of *B. lactis* on apple chips [13], as well as of *L. plantarum* on apple and melon pieces, were observed [18]. These studies reported changes in color (darkening) and pH and decreased sensory acceptance of probiotic-treated fruit. In this context, the incorporation of probiotics into edible films/coatings should decrease or neutralize the negative impacts related to the probiotic activity on the physicochemical and/or sensory characteristics of coated fruit and vegetables [9].

The application of probiotic-loaded edible films/coatings must be effective in controlling fruit and vegetable ripeness due to decreased gas diffusion, dehydration (and resulting softening changes), respiration rate, oxidation, carbohydrate hydrolysis and other metabolic activities linked to the senescence process in coated products. The application of probiotic-loaded edible films/coatings has also been effective to reduce water vapor permeability, oxygen diffusion and light transmittance in fruit and vegetables, hindering possible deterioration due to the lipid oxidation [25,28]. The addition of probiotics to the film/coating-forming solution improves the barrier performance of the film/coating due to the intermolecular interactions (e.g., hydrogen bonds) between the probiotic cells and polymeric matrix, reducing the inter-molecular distance among them [28]. Furthermore, the long path produced by probiotic aggregates distributed in the matrix slows the movement of molecules through the film/coating [45].

These effects could help coated fruit and vegetables to keep freshness, quality and nutritional characteristics for a more prolonged storage time [13,19,23]. The effects of the application of probiotic-loaded edible films/coatings on overall quality and sensory parameters of fruit and vegetables in different studies are shown in Table 4.

The application of a konjac gum coating loaded with probiotic *L. plantarum* decreased the color changes and preserved more chlorophyll and ascorbic acid in kiwi slices during refrigeration storage when compared to konjac gum coating without probiotic [2]. The application of alginate coatings containing linseed mucilage, FOS and *L. casei* LC-01 helped to preserve the physicochemical characteristics of minimally processed yacon, reducing the weight loss and contributing to the maintenance of soluble solids and acidity during refrigeration storage, being indicative of lowered physiological activity in coated product. The use of linseed mucilage in probiotic-loaded alginate coatings contributed to reducing the darkening in fresh-cut yacon [20].

The application of carboxymethylcellulose coating loaded with *L. plantarum* improved the physicochemical characteristics of strawberries, reducing the weight and ascorbic acid losses during refrigeration storage without affecting negatively the sensory characteristics of coated fruit [25]. The use of alginate and gelatin coatings counteracted the negative browning effects of the direct application of *B. lactis* on apple chips, besides increasing the sensory acceptance for a prolonged storage period [14].

The application of probiotic-loaded edible films/coatings has also been effective to keep high contents of phenolic compounds and antioxidant capacity of coated fruit and vegetables, which has been linked to the contents of different phenolic compounds, carotenoids and vitamins. The application of probiotic-loaded films/coatings could decrease the loss of phenolic compounds and protect coated fruit and vegetables from oxidative damage and accumulation of free radicals, retarding the product senescence [46]. Furthermore, probiotic-loaded edible films/coatings could avoid enzymatic oxidation of phenolic compounds in coated fruit and vegetables, causing protective effects against oxidation [2].

The improvements in antioxidant capacity in fruit and vegetables treated with probiotic-loaded edible films/coatings have also been related to the production of specific compounds (e.g., exopolysaccharides) by metabolically active probiotic cells during storage [28]. Different probiotics have produced exopolysaccharides with strong antioxidant capacity and potential to be exploited to protect fresh fruit and vegetables from oxidative damage during storage [47]. The application of a gelatin coating loaded with *L. rhamnosus* improved the antioxidant capacity of strawberries during 16 days of refrigeration storage [12]. The application of konjac gum coating loaded with *L. plantarum* caused higher DPPH scavenging activities and total phenolic contents in fresh-cut kiwi during cold storage [2]. The loading of *L. plantarum* and *P. pentosaceus* caused increased antioxidant capacity of a cassava starch coating applied to banana in a dose-dependent manner [28]. These results were attributed to the exopolysaccharides with strong scavenging hydroxyl radical ability produced by LAB loaded into the cassava starch coating [48]. Additionally, exopolysaccharides can enhance the activity of the intracellular antioxidant enzyme system, inhibit the lipid peroxidation and keep the cell integrity of probiotics in edible films/coatings [28].

## 6. Conclusions

The results of the available studies have indicated the application of probiotic-loaded edible films/coatings as a simple and affordable bioprotection technique with a great potential for application in fresh and minimally processed fruit and vegetables not requiring the use of expensive ingredients and laboratory equipment. The application of probiotic-loaded edible films/coatings could cause improvements or maintenance of parameters linked to the quality, safety, functionality and storage stability of fresh and minimally processed fruit and vegetables. Most of the studies have investigated the use of polysaccharides as a material to carry probiotics in fresh and minimally processed fruit and vegetables, although proteins have been shown as compatible material to provide higher survival of probiotics in edible films/coatings when compared to polysaccharides. Some factors related to the examined probiotics strains could be important to the successful application of these composite materials, such as their survival rate, loaded dose and production of antimicrobial metabolites under controlled conditions found in fruit and vegetables intended for their application. Despite the promising results found in the available literature regarding the use of probiotic-loaded edible films/coatings for fruit and vegetable bioprotection, further studies are needed to elucidate different influential factors to optimize the survival of probiotics in the formulated composite materials when applied to fruit and vegetables, including the selection of probiotic strains with strong and wide-spectrum antagonistic properties, materials (alone or in combination) and processing conditions used for film/coating formulation and storage conditions. Furthermore, the investigation of the effects of these variables on parameters related to the safety, quality and acceptability of coated fruit and vegetables must also be considered.

## Figures and Tables

**Table 1:** Materials used to formulate probiotic-loaded edible films/coatings applied to fresh or minimally processed fruit and vegetables.

Probiotic Bacteria	Materials Used to Formulate Edible Coating/Films	Additives	Fruit/Vegetable	Reference
*Lacticaseibacillus casei*	Alginate	Glycerol (5 g/L); polysorbate 80 (1 g/L); linseed mucilage (0.3° Brix); FOS (15 g/L); calcium chloride solution (20 g/L)	Fresh-cut yacon	[20]
*Lactobacillus acidophilus*	Alginate solution	Glycerol (0.5 g); sunflower oil (0.075 g); Tween 80 (0.025 g); bivalent Ca^2+^ ion	Minimally processed carrot	[21]
*Lactiplantibacillus plantarum*	Alginate + chitosan	Citrate (0.2%, *w*/*v*), ascorbate (1% *w*/*v*), CaCl_2_ (0.5%, *w*/*v*)	Apple and melon pieces	[18]
*Lacticaseibacillus rhamnosus*	Alginate + prebiotics	Glycerol (15 g/kg); inulin andoligofructose (40 and 80 g/kg); CaCl_2_ (20 g/kg)	Fresh blueberry	[22]
*L. casei*	Whey protein isolate	Glycerol (5%, *w*/*v*)	Cherry tomato and Thompson grape	[23]
*L. rhamnosus*	Alginate + prebiotic	Glycerol (1%); inulin (non-detailed concentration); CaCl_2_ (2% *w*/*v*)	Minimally processed apple	[24]
*L. plantarum*	Carboxymethylcellulose	Glycerol 30% (*w*/*w*)	Strawberry	[25]
*L. plantarum*	Pregelatinized potato starch or sodium caseinate	Oleic acid (ratio 0.1:1)	Grape	[26]
*L. acidophilus*	Sodium alginate	Glycerol (0.75 g); sunflower oil (0.04 g), Tween 80 (0.05 g); coconut water (70%)	Minimally processed carrot	[27]
*Bifidobacterium animalis* subsp. *lactis*	Alginate + gelatin	CaCl_2_ (0.5%, *w*/*v*)	Apple pieces	[14]
*Lacticaseibacillus paracasei*	Corn starch + gelatin	Glycerol (1%, *w*/*w*); Gamma radiation (3.5 KGy)	Tomato	[6]
*L. plantarum*, *Pediococcus pentosaceus*	Cassava starch	Glycerol (1.5%, *w*/*w*); carboxymethylcellulose (0.2%, *w*/*w*)	Banana	[28]
*L. rhamnosus, B.* *l* *actis*	Alginate + prebiotics	Glycerol (1.5%, *w*/*w*); inulin (8%, *w*/*w*) and oligofructose (8%, *w*/*w*); CaCl_2_ (2%, *w*/*v*)	Fresh-cut apple	[29]
*L. plantarum* AF1, *L. plantarum* LU5, *L. plantarum* LP3	Konjac glucomannan (gum)	Glycerol (25%, *w*/*w*)	Fresh-cut kiwi	[2]
*L. rhamnosus*	Gelatin + prebiotic	Glycerol (15%, *w*/*w*); inulin (2.5%, *w*/*w*)	Strawberry	[12]

NP: Data not provided; FOS: Fructooligosaccharide; CaCl_2_: Calcium chloride.

**Table 2:** Viable counts (survival) of probiotics loaded into edible films/coatings applied to fruit and vegetables.

Probiotic Bacteria	Materials and Concentrations Used to Formulate Edible Coating/Films	Initial Inoculum (log CFU/g or mL)	Final Viable Counts (log CFU/g or mL)	Storage Condition	Reference
*Lacticaseibacilluscasei* LC-01	Alginate (20 g/L)	8–9	8.0–8.7	5 °C, 15 days	[20]
*Lactobacillus acidophilus* La-14	Alginate solution (1.75%, *w*/*w*)	7.36	7.1	8 °C, 19 days	[21]
*Lactipnatibacillus plantarum* c19	Alginate (2%, *w*/*v*) + chitosan (1%, *w*/*v*)	6.8	4.5–5.3 (chitosan) and 6.7–7.3 (alginate)	4 °C, 14 days	[18]
*Lacticaseibacillusrhamnosus* CECT8361	Alginate (20 g/kg) + prebiotics	7.1–7.6	5 (without prebiotic) and 6.2 (with prebiotic)	5 °C, 21 days	[22]
*L. casei* 01	Whey protein isolate (10%, *w*/*v*)	7.8	5.7	25 °C, 28 days	[23]
*L. rhamnosus* B-445	Alginate (2%, *w*/*v*) + prebiotic	8.22–8.34	6.0–7.4	5 °C, 13 days	[24]
*L. plantarum* PTCC1058	Carboxymethylcellulose (1%, *w*/*v*)	6.52–8.90	5.3–8.4	4 °C, 15 days	[25]
*L. plantarum*	Pregelatinized potato starch (2%, *w*/*v*) or sodium caseinate (2%, *w*/*v*)	7.7	4.1–5.2 (pregelatinized potato starch); 6.1–6.2 g (sodium caseinate)	20 °C, 7 or 9 days	[26]
*L. acidophilus* LA3	Sodium alginate (1.5%, *w*/*w*)	9	<4 (alginate/prebiotic); 6.3 (alginate/coconut water/prebiotic)	8 °C, 21 days	[27]
*Bifidobacterium animalis* subsp. *lactis* DSM10140	Alginate (2%, *w*/*v*) or gelatin (sucrose 0.5%—*w*/*v*, corn starch 0.08%—*w*/*v*, lemon juice 0.05%—*v*/*v*)	8	8.0–6.8 (alginate coated, 8 °C) *	4 and 8 °C, 10 days	[14]
*Lacticaseibacillus paracasei*	Corn starch (4 g/mL) + gelatin (1 g/mL)	NP	NP	5 °C, 14 days	[6]
*L. plantarum* and *Pediococcus pentosaceus*	Cassava starch (4%, *w*/*w*)	~8 and 9	~7 and 8	30 °C, 48 h (drying)	[28]
*L.rhamnosus* CECT 8361, *B. lactis* CECT 8145	Alginate (2%, *w*/*w*) + prebiotics	11.7	9.1–9.5	5 °C, 8 days	[29]
*L. plantarum* AF1, *L. plantarum* LU5, *L. plantarum* LP3	Konjac glucomannan (6%, *w*/*w*)	9.4	6.4–7.1	4 °C, 5 days	[2]
*L. rhamnosus* HN001	Gelatin (5%, *w*/*w*) + prebiotic	11	7.0–7.4 (with prebiotic compounds)	4 °C, 16 days	[12]

* Gelatin coating did not exert a negative effect on the viable counts. NP: Data not provided.

**Table 3:** Antimicrobial effects of probiotic-loaded edible films/coatings when applied to fresh and minimally processed fruit and vegetables.

Probiotics	Target Microorganism/Microbial Group	Fruit/Vegetables	Main Effects of Probiotic Coating	Reference
*Lacticaseibacillus casei*	NP	Fresh-cut yacon	NP	[20]
*Lactobacillus acidophilus*	Aerobic mesophilic bacteira; molds and yeasts	Minimally processed carrot	Inhibited the fungal growth during the 19 days of storage. Uncoated fruit had higher levels of mold and yeast contamination at the end of the storage period.	[21]
*Lactiplantibacillus plantarum*	Molds and yeasts and psychrotrophic bacteria	Apple and melon pieces	Counts of molds, yeasts and psychrotrophic bacteria were below the limit of detection during the storage.	[18]
*Lacticaseibacillus rhamnosus*	Native microbiota; *Listeria innocua* and *Escherichia coli* O157:H7	Fresh-blueberry	Counts of native microbiota remained at safe levels during refrigeration storage. Reduction of counts of *L. innocua* up to 1.7 log units.	[22]
*L. casei*	NP	Cherry tomatoes and grape	NP	[23]
*L. rhamnosus*	Mesophilic bacteria and molds and yeasts	Minimally processed apple	Counts of mesophilic bacteria, molds and yeast were reduced, extending the shelf life of fresh-cut apples.	[24]
*L. plantarum*	Molds and yeasts	Strawberry	Reduction of mold and yeast counts on strawberries. Inverse correlation between the number of viable probiotic cells and population of molds and yeasts, indicating a dose-dependent effect.	[25]
*L. plantarum*	*Botrytis cinerea*	Grape	Reduction of incidence and severity of *B. cinerea* infection. Potato starch-based formulation without oleic acid reduced the *B. cinerea* incidence more than when applied in sodium caseinate formulation or in water.	[26]
*L. acidophilus*	Thermotolerant coliforms, molds and yeasts, *Salmonella* spp.	Minimally processed carrot	Carrot submitted to the different treatments had absence of thermotolerant coliforms, *Salmonella* spp. and molds and yeasts during storage.	[27]
*Bifidobacterium animalis subsp. lactis*	NP	Apple pieces	NP	[14]
*Lacticaseibacillus paracasei*	Native microbiota	Tomato	Coated tomato had the lowest percentage of rot and bacterial counts at the end of the storage period, which were attributed to the effects of gamma irradiation increasing the antimicrobial activity of irradiated lactic acid bacteria supernatant.	[6]
*L. plantarum*, *Pediococcus pentosaceus*	NP	Banana	NP	[28]
*L. rhamnosus, B.* *lactis*	*E. coli* O157:H7; *L. innocua;* molds and yeasts	Fresh-cut apple	Maintenance of the microbiological quality of coated apples.	[29]
*L. plantarum* AF1, *L. plantarum* LU5, *L. plantarum* LP3	Molds and yeasts	Fresh-cut kiwi	Coated kiwi slices had reduced mold and yeast counts.	[2]
*L. rhamnosus*	Molds and yeasts; aerobic mesophilic bacteria	Strawberry	Coated strawberries had reduced counts of mesophilic bacteria and molds and yeasts.	[12]

NP: Data not provided.

**Table 4:** Effects of the application of probiotic-loaded edible films/coatings on quality (physicochemical and/or sensory) parameters of fruit and vegetables.

Probiotic Bacteria	Coating/Film	Fruit/Vegetable	Main Effects Related to Physicochemical Parameters	Main Effects Related to Sensory Parameters	Reference
*Lacticaseibacillus casei* LC-01	Alginate, linseed mucilage, fructooligosaccharides	Fresh-cut yacon	Coated yacon had reduced weight loss and maintained the acidity and soluble solids contents during refrigeration storage.	Coated fruit had decreased darkening.	[20]
*Lactobacillus acidophilus*	Alginate solution	Minimally processed carrot	Coated carrot had reduced metabolism, with less variation in acidity, and maintained the moisture content during refrigeration storage.	Coated fruit had decreased color change (white surface discoloration).	[21]
*Lactiplantibacillus plantarum*	Alginate powder or chitosan	Apple and melon pieces	Alginate coating caused higher probiotic survival on fruit, and decreased the negative effects of the probiotic-loaded coatings on color and pH of fruit during refrigeration storage.	NP	[18]
*Lacticaseibacillus rhamnosus*	Alginate, prebiotic	Fresh blueberry	Coating had no effects on instrumental firmness and color of fruit.	Coated fruit had satisfactory visual quality, odor and flavor, being sensorially acceptable up to day 14 of refrigeration storage.	[22]
*L. casei*	Whey protein isolate	Cherry tomato, Thompson grape	Coated grape and tomato had delayed ripening evolution. High probiotic viable counts on coated fruit were found for up to 14 days of room temperature storage.	NP	[23]
*L. rhamnosus*	Alginate, prebiotic	Minimally processed apple	Coated apple maintained the moisture content, total soluble solids, firmness, ascorbic acid, pH and titratable acidity during refrigeration storage.	Color, odor, taste and texture characteristics of coated fruit were maintained up to 13 days of storage.	[24]
*L. plantarum*	Carboxymethylcellulose	Strawberries	Coating had positive effects on the physicochemical parameters of strawberries, reducing the weight loss and slowing down the deterioration rate of ascorbic acid and phenolic compounds during refrigeration storage.	Sensory characteristics of coated fruit were not affected, which were acceptable in terms of color, flavor, taste, texture and overall acceptability during storage.	[25]
*L. plantarum*	Pregelatinized potato starch or sodium caseinate	Grape	Coatings had little effect on weight, color, firmness and soluble solids contents of grapes during room temperature storage.	NP	[26]
*L. acidophilus*	Sodium alginate	Minimally processed carrot	NP	Improvement of the sensory attributes of coated fruit, particularly of color, appearance and texture.	[27]
*Bifidobacterium animalis* subsp. *lactis*	Alginate, gelatin	Apple chips	Addition of isolated probiotic caused worsening of color of apple chips, with an increase in browning index. Probiotic-loaded coating counteracted this negative effect.	Coated apple pieces had higher sensory scores and lower browning index after 10 days of refrigeration storage.	[14]
*Lacticaseibacillus paracasei*	Corn starch, gelatin	Tomato	Coated tomato had decreased weight loss and decay percentage, and higher ascorbic acid, lycopene, total sugars and total phenolic contents.	NP	[6]
*L. plantarum, Pediococcus pentosaceus*	Cassava starch	Banana	Coated banana had prolonged shelf life and reduced black spot development for up to 7 days of storage.	NP	[28]
*L. rhamnosus, B. lactis*	Alginate, prebiotic	Fresh-cut apple	Coated apple maintained the total phenolic contents and antioxidant capacities during refrigeration storage.	Apple coated with prebiotic and *B. lactis* remained sensorially acceptable up to 8 days of storage	[29]
*L. plantarum*	Konjac glucomannan	Fresh-cut kiwi	Coated kiwi had decreased decay and color changes, higher total phenolic content and antioxidant capacities, and maintained chlorophyll and ascorbic acid contents during refrigeration storage.	Probiotic treatments positively influenced the overall acceptability of fruit, while uncoated fruit were rejected.	[2]
*L. rhamnosus*	Gelatin, prebiotic	Strawberry	Coated strawberry had decreased weight loss and preserved the total phenolic contents and antioxidant capacity during refrigeration storage.	NP	[12]
